# Do ScvO_2_ variations induced by passive leg raising predict fluid responsiveness? A prospective study

**DOI:** 10.14814/phy2.15012

**Published:** 2021-09-07

**Authors:** Raphaël Giraud, Bojana Vujovic, Benjamin Assouline, Ivo Neto Silva, Karim Bendjelid

**Affiliations:** ^1^ Intensive Care Unit Geneva University Hospitals Geneva Switzerland; ^2^ Faculty of Medicine University of Geneva Geneva Switzerland; ^3^ Geneva Hemodynamic Research Group University of Geneva Geneva Switzerland

**Keywords:** cardiac output, central venous oxygen saturation, echocardiography, fluid responsiveness, hemodynamic, passive leg raising, ScvO_2_

## Abstract

**Objective:**

The present study investigates whether ScvO_2_ variations induced by passive leg raising (PLR) are able to predict fluid responsiveness (FR) in mechanically ventilated patients.

**Design:**

A monocentric prospective clinical study.

**Setting:**

An intensive care division in a tertiary hospital.

**Patients:**

The inclusion criteria were elective postoperative cardiac surgery patients who were over 18 years old, deeply sedated, mechanically ventilated and needed volume expansion (VE). Fluid responders (R) were defined as patients who increased their left ventricular outflow tract velocity time integral (VTI) ≥15% after VE.

**Intervention:**

In patients included in this study, continuous ScvO_2_ monitoring (CeVOX device, Pulsion Medical Systems) and VTI (transthoracic echocardiography) were measured simultaneously before and during a PLR test and before and after VE (with 500 ml of saline).

**Measurements and main results:**

Thirty‐three consecutive patients were included in this study. In 15 patients with a positive PLR test (increase in VTI ≥15%), ScvO_2_ increased during PLR by 9 ± 4%. In the 18 patients with a negative PLR test, ScvO_2_ did not significantly change during PLR. VE increased ScvO_2_ by 9 ± 6% and 2 ± 4% in responders and nonresponders, respectively. If ScvO_2_ increased by >4% during the PLR test, then a positive VTI response (≥15%) was diagnosed with a sensitivity of 93% (68–99%) and a specificity of 94% (63–99%) (Area under the receiver operating characteristic curve 0.92 ± 0.58, *p *< 0.05). Moreover, ScvO_2_ variations were able to distinguish responders to VE from nonresponders to VE with a sensitivity of 87% (68–99%) and a specificity of 89% (63–99%) (Area under the receiver operating characteristic curve 0.89 ± 0.07, *p *< 0.05).

**Conclusions:**

ScvO_2_ variation induced by PLR is a reliable, minimally invasive parameter for predicting FR at the postoperative cardiac surgery bedside of mechanically ventilated, critically ill patients.

## Graphical abstract

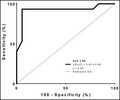

## 1 INTRODUCTION

Volume expansion (VE) is often considered the first‐line therapy for increasing cardiac output (CO) in patients with circulatory failure, especially in the postoperative period of cardiac surgery, where most of these patients suffer from hypovolemia (Bendjelid et al., 2006; Giraud, Siegenthaler, Gayet‐Ageron, et al., 2011). Therefore, it is crucial to predict the response of CO to fluid infusion before performing the challenge (Ganter et al., 2018), especially when the preload responsiveness is not obvious, as is the case in the postoperative phase of cardiac surgery.

The passive leg raising (PLR) test, a mechanical method, is one of the approaches currently available for this purpose (Monnet et al., 2006). Transthoracic echocardiography (TTE) is also able for evaluating the hemodynamic response to both PLR and VE using the velocity time integral of aortic blood flow measurement (VTIAo) changes (Lamia et al., 2007). Moreover, for decades, mixed venous oxygen saturation (SvO_2_) monitoring has been used as a substitute marker of oxygen delivery‐consumption coupling during the treatment of critically ill patients (Kandel & Aberman, 1983). From a physiological point of view, parallel increases in CO, SvO_2_, and ScvO_2_ after VE (ΔScvO_2_) exist (Giraud, Siegenthaler, Gayet‐Ageron, et al., 2011). Indeed, we have already demonstrated that ScvO_2_ variations after VE are able to categorize VE efficiently as an alternative marker to define fluid responsiveness (Giraud, Siegenthaler, Gayet‐Ageron, et al., 2011). As the continuous assessment of ScvO_2_ with the CeVOX device (Pulsion Medical Systems, Munich, Germany) is able to track ScvO_2_ variation (Baulig et al., 2008; Herner et al., 2018), the aim of the present prospective study is to evaluate whether ΔScvO_2_ induced by PLR could be a valuable minimally invasive tool for predicting the potential positive hemodynamic response of VE (fluid responsiveness) after cardiac surgery.

## 2 MATERIALS AND METHODS

### 2.1 Study design

This study was carried out after approval was obtained from the institutional ethical review board at Geneva University Hospitals (NAC 11–055). All patients and/or their surrogates agreed to participate in the study by signing a written informed consent.

### 2.2 Study population

The study was conducted in a tertiary care university hospital in Geneva. The included patients were over 18 years old, deeply sedated, mechanically ventilated, and needed VE after elective cardiac surgery, as requested by the physician in charge; in addition, they were equipped with a triple lumen central venous catheter (CVC) (standard of care). Patients with bad echogenicity for whom the measurement of VTIAo was not possible, with hypothermia (Bendjelid et al., 2006; Giraud et al., 2013) who had active bleeding and/or who were expected to survive less than 24 hours were excluded. Patients were recruited during a 1‐year period. During the study protocol, body temperature, the ventilation settings, catecholamine dosing and analgesia‐sedation remained unchanged. The decision to administer a fluid bolus for volume expansion (500 ml of 0.9% saline in a 10‐min infusion in the context of standard of care) was left to the decision of the attending intensivist.

The attending intensivist's decision to realize a VE was a prerequisite for the inclusion of the patient in the study. All patients meeting the inclusion criteria were included in the study unless they refused to participate or the principal investigators were not available.

### 2.3 Hemodynamic monitoring

In all patients, standard monitoring (IntelliVue MP70, Philips Medical Systems, Philips Healthcare, the Netherlands) was used, which included 5‐lead ECG, pulse oximetry, and continuous central venous pressure (CVP) via a central venous catheter (CVC, Arrow International Inc, Teleflex Medical AG, Belp, Switzerland). Invasive arterial blood pressure was assessed via left radial artery access by a 3‐French catheter (Vygon, Niederwangen, Bern, Switzerland). Immediately after the inclusion of a patient, a CeVOX probe (PV2022‐37; Pulsion Medical System, Getinge, Munich, Germany) was inserted in the medial lumen of the CVC, ending at the tip of the catheter according to the manufacturer´s recommendations. This provided the withdrawal of blood at the most distal lumen of the CVC in close proximity to the fiber probe. The position of the tip was controlled (and corrected) according to x‐ray.

For blood gas analysis, a Radiometer ABL 90 flex blood gas analyzer (Radiometer RSCH GmbH, Thalwil, Switzerland) was used. Baseline BGA was performed to calibrate the CeVOX subunit of a PiCCO_2_ monitor (Pulsion Medical Systems, Getinge, Munich, Germany). No further calibrations of CeVOX were performed during the study period. After this calibration, when patients were in the semi‐recumbent position, we collected demographic characteristics and hemodynamic variables, including heart rate, arterial, and central venous pressures and ScvO_2_ values (CeVOX). The pressure sensors connected to the arterial and central venous catheters were fixed on the upper arm of the patient at the estimated level of the right atrium. The echocardiographic examination was performed by the same trained operators (RG and BV) using a transthoracic ultrasound device (Philips Sparq Ultrasound System, Gland, Switzerland) equipped with a tissue Doppler imaging program and an S4–2 cardiac sector probe [2–4 MHz].

Conventional echocardiography, including M‐mode, two‐dimensional analysis, and Doppler measurements, was performed. Echocardiographic images were recorded together with the electrocardiogram. All measurements were recorded at a speed of 150 mm/s and were stored digitally in the hardware for later playback and analysis. All measurements were evaluated by two physicians (RG and BV). These two investigators were not aware of ScvO_2_ values while measuring VTIAo. Using the apical five‐chamber view, the VTIAo was computed from the area under the envelope of the pulsed‐wave Doppler signal (laminar flow signal) obtained at the level of the aortic annulus. The VTIAo value was averaged over 5–10 consecutive measurements in sinus rhythm or paced patients and over 10 measurements in patients with atrial fibrillation.

**FIGURE 1:**
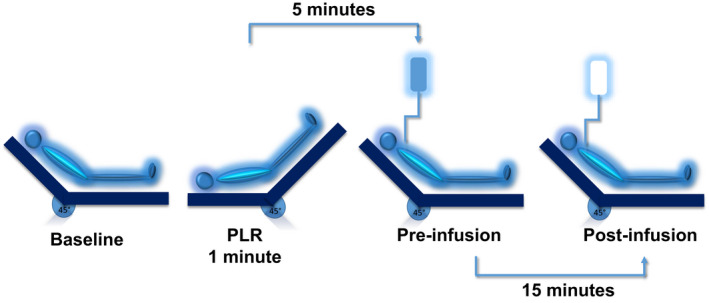
Study protocol

A PLR test was then performed by transferring the patient to the PLR position, in which the lower limbs were passively elevated at 45° and the trunk was horizontal (bed repositioning) (Monnet & Teboul, 2015). When the PLR test had induced its maximal effect, which habitually occurs within 1 min, we performed another set of measurements, including mean arterial pressure, heart rate, central venous pressure, VTIAo measured by TTE and ScvO_2_ (CeVOX), systematically with the present sequence. Then, we moved the patient back to the semi‐recumbent position. After 5 min, we performed a third set of measurements, including heart rate, arterial and central venous pressures, and VTIAo by TTE. As the PLR test was planned in view of infusing fluid, VE with 500 ml of saline was administered over a period of 10 min. Immediately after the end of the fluid infusion, the last set of measurements of mean arterial pressure, heart rate, central venous pressure, ScvO_2_ (CeVOX), and VTIAo by TTE was performed (Figure 1). Catecholamine and sedative drug doses and ventilation settings were kept constant during the study.

### 2.4 Statistical analysis

The PLR test was defined as positive if it increased VTIAo ≥15%. A positive response to VE was defined if VTIAo increased ≥15% just after fluid administration. ΔScvO_2_ in percent was defined as ScvO_2PLR_ – ScvO_2Baseline_/ScvO_2Baseline_ and ScvO_2VE_ – ScvO2_Preinfusion_/ScvO_2Preinfusion_. Data are expressed as the mean ± standard deviation (SD), median [interquartile range, IQR], and number (percentage). Normality was assessed by the Kolmogorov–Smirnov test. Pairwise comparisons of values between different study times were performed by paired Student *t*‐tests. Comparisons between patients with positive PLR and patients with negative PLR tests were performed by two‐tailed Student *t*‐tests or the Wilcoxon test, as appropriate. We compared the relative changes in VTIAo to ScvO_2_ variations by linear regression analysis (for percent changes). Correlations were assessed by the Pearson coefficient. Receiver operating characteristic (ROC) curves (with 95% confidence intervals) were generated to describe the ability of the PLR‐ and VE‐induced percent changes in ScvO_2_ to detect the PLR‐ and VE‐induced percent changes in VTIAo. The areas under ROC curves were compared by the Hanley‐McNeil test (Hanley & McNeil, 1982). The Youden index was calculated as sensitivity +specificity −1 and was used to determine the diagnostic threshold.

Statistical analyses were performed using GraphPad Prism (GraphPad Prism version 7.00 for Windows, GraphPad Software, La Jolla, California USA).

## 3 RESULTS

We initially recruited 38 patients for the study. Five patients were excluded because of a poor echocardiographic window without the possibility of measuring the VTIAo. Consequently, 33 patients were included in the study. The demographics, pathology, and surgical interventions are presented in Table 1. No patient had significant aortic or mitral stenosis or regurgitation after the surgery. Seventeen patients had ischemic cardiomyopathy and underwent a coronary artery bypass graft (CABG) (52%), while 13 patients (39%) had valve diseases (eight underwent aortic valve replacement and five mitral valve surgeries). Other cardiac diseases included combined surgeries (CABG +aortic replacement, Bentall or Tirone David surgeries). Twenty patients (61%) were in synchronized intermittent mandatory ventilation (SIMV) mode, while 13 were in pressure support (PS) mode (39%). All patients were sedated with propofol and fentanyl at 277 ± 65 mg/h and 64 ± 29 µg/min, respectively. All patients had vasoactive support with norepinephrine, and 15 patients (45%) had inotropic support with dobutamine. The baseline respiratory, hemodynamic, and medication characteristics are summarized in Table 2.

**TABLE 1:** Patient characteristics

	N = 33
Age (years)	63 ± 12
Gender M/F	18/15
Weight, kg	78 ± 17
Body mass index (kg/m^2^)	27 ± 5
Pathology	
Valvular cardiomyoapthy (%)	13 (39)
Ischemic cardiomyopathy (%)	17 (52)
Other cardiomyopathy	5 (15)
Surgical intervention	
Aortic valve replacement	8 (24)
Mitral valve surgery (plasty or replacement)	5 (15)
Coronary Artery Bypass Graft Surgery	17 (52)
Other cardiac surgery	5 (15)

**TABLE 2:** Baseline parameters

	N = 33
Ventilatory mode (%)	13 PS (39) / 20 SIMV (61)
Tidal volume (mL)	484 ± 90
Respiratory rate (b/min)	15 ± 4
PEEP (cmH_2_O)	6 ± 2
PROPOFOL (mg/h)	277 ± 65
FENTANYL (µg/h)	64 ± 29
NOREPINEPHRINE (µg/kg/min)	0.06 ± 0.04
DOBUTAMINE (µg/kg/min)	4 ± 3
Lactate (mmol/L)	1.5 ± 0.5
HR (/min)	78 ± 10
MAP (mmHg)	70 ± 10
CVP (mmHg)	8 ± 4
VTI (cm)	15.5 ± 3.9
ScvO_2_ (%)	65 ± 8

PS, Pressure support; PEEP, Positive End‐Expiratory Pressure; VTI, Velocity Time Integrale; HR, Heart Rate; MAP, Mean Arterial Pressure; CVP, Central Venous Pressure; ScvO_2_, Central venous Oxygen Saturation.

### 3.1 Effects of PLR and volume expansion on ScvO_2_

The PLR test was positive (PLR‐induced increase in CI ≥15%) in 15 patients (45%), with an increase in VTIAo of at least 15%. (Table 3). In all the patients classified as potential fluid responders, VTIAo and ScvO_2_ were significantly increased by 24 ± 4% and 9 ± 7%, respectively (*p *< 0.05 for both). ΔScvO_2_ correlated with ΔVTI (r^2^ = 0.44, *p *< 0.001) following PLR.

**TABLE 3:** Differences between responders and nonresponders

	R (N = 15)	NR (N = 18)	*p*
Ventilatory mode	3 PS / 12 SIMV	8 PS / 10 SIMV	
Tidal volume (mL)	481 ± 116	486 ± 65	0.89
Respiratory rate (b/min)	15 ± 2	16 ± 5	0.31
PEEP (cmH_2_O)	6 ± 1	7 ± 1	0.33
NOREPINEPHRINE (µg/kg/min)	0.05 ± 0.03	0.06 ± 0.04	0.37
DOBUTAMINE (µg/kg/min)	3 ± 2	4 ± 4	0.5
VTI (cm)	15 ± 4	16 ± 4	0.58
HR (/min)	75 ± 8	79 ± 11	0.25
MAP (mmHg)	68 ± 9	72 ± 11	0.24
CVP (mmHg)	7 ± 3	9 ± 4	0.28
Baseline ScvO_2_ (%)	63 ± 10	67 ± 6	0.12

PS, Pressure support; SIMV, Synchronized Intermittent Mandatory Ventilation; PEEP, Positive End‐Expiratory Pressure; VTI, Velocity Time Integral; HR, Heart Rate; MAP, Mean Arterial Pressure; CVP, Central Venous Pressure; ScvO_2_, Central Venous Oxygen Saturation.

In the 18 patients for whom the PLR test was negative (VTIAo increased by <15%), VTIAo and ScvO_2_ did not significantly change during PLR (4 ± 8% and 2 ± 4%, respectively). ΔScvO_2_ correlated with VTI changes following VE (r^2^ = 0.31, *p *< 0.001; additional file, Figures S1 and S2, respectively).

### 3.2 Ability of ScvO_2_ changes to predict fluid responsiveness after a positive PLR test

**FIGURE 2:**
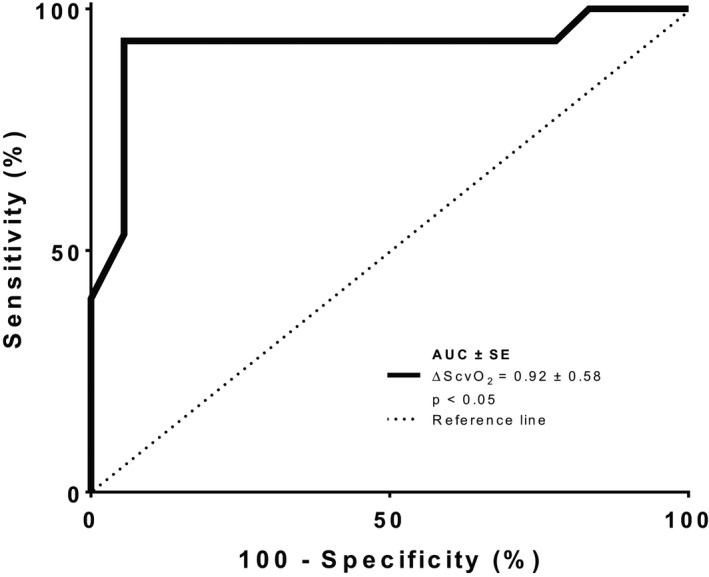
ROC curves comparing the ability of ScvO_2_ variations to discriminate responders (CI increases ≥15% after volume expansion and nonresponders (CI increases <15% after PLR)

**FIGURE 3:**
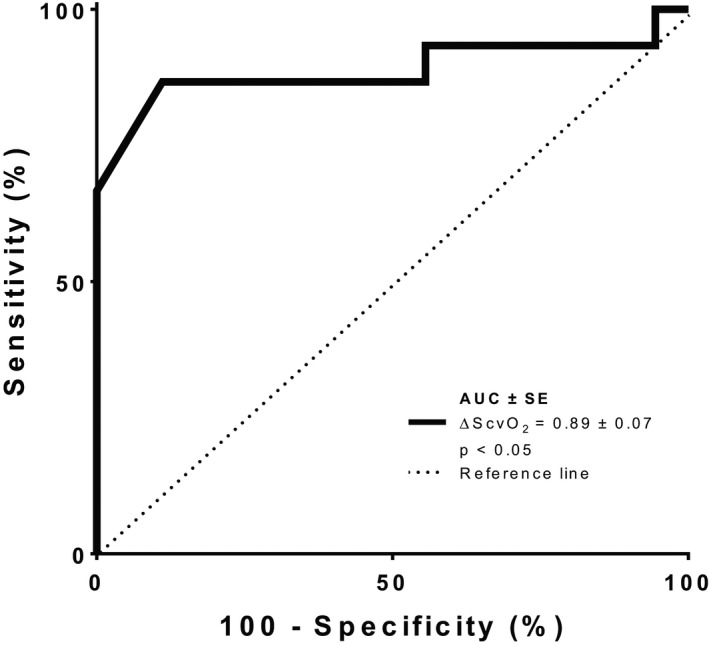
ROC curves comparing the ability of ScvO_2_ variations to discriminate responders (CI increases ≥15% after volume expansion and nonresponders (CI increases <15% after VE)

During the PLR test, if ScvO_2_ increased by >4% following the maneuver, a positive VTI response (≥ 15%) was diagnosed with a sensitivity of 93% (68–99%) and a specificity of 94% (63–99%) (Area under the receiver operating characteristic curve 0.92 ± 0.58, *p *< 0.05) (Figure 2). Additionally, a ScvO_2_ increase of >4.5% following VE discriminated responders (CI increases ≥15% after VE) from nonresponders with a sensitivity of 87% (68–99%) and a specificity of 89% (63–99%) (Area under the receiver operating characteristic curve 0.89 ± 0.07, *p *< 0.05) (Figure 3).

## 4 DISCUSSION

The present study emphasizes that PLR‐induced ScvO_2_ changes measured continuously by the CeVOX optical fiber are a reliable minimally invasive parameter that is able to predict a potential fluid‐responsive patient before VE. This result is demonstrated in mechanically ventilated patients undergoing cardiac surgery, specifically patients for whom it is difficult to assess preload dependency due to their transitory capillary leak state.

Approximately half of the patients included in the current study were fluid responsive, which is consistent with the literature (Cannesson et al., 2011; Marik et al., 2009). The PLR maneuver is an easy and reliable test to predict fluid responsiveness (Mesquida et al., 2017; Monnet et al., 2016) and is now accepted in clinical practice (Cecconi et al., 2014; Cherpanath et al., 2016; Monnet et al., 2016). Nevertheless, its main drawback is that, like the fluid challenge (Monnet et al., 2011; Pierrakos et al., 2012), its effects must be assessed by the direct measurement of CO, which must be precise and able to detect short‐term changes with precision (Giraud et al., 2011b, 2017; Monnet & Teboul, 2015). The present study suggests that the changes in ScvO_2_ value during continuous monitoring might be used as a surrogate for the changes in CO during PLR and could then be used to assess preload responsiveness with acceptable accuracy. Indeed, following the PLR maneuver, the majority of patients who were classified as responders to PLR were responders in terms of cardiac output increase after VE.

Because ScvO_2_ is a metabolic parameter, it is influenced by not only cardiac output, but also by arterial oxygen saturation (SaO_2_), oxygen consumption (VO_2_), and hemoglobin concentration. Therefore, changes in ScvO_2_ are directly proportional to those in CO only when these three parameters remain constant, as it was the case of our postoperative patients. Indeed, in shocked patients increase in cardiac output which is followed by an increase in oxygen delivery will, first off all, increase VO_2_, without changes in ScvO_2_ value. We found that although ΔSvO_2_ exhibited a correlation with ΔScvO_2_, the absolute values of ΔSvO_2_ were smaller than those of ΔScvO2 (Giraud, Siegenthaler, Gayet‐Ageron, et al., 2011). Moreover, Monnet et al reported no significant changes in ScvO_2_ in fluid responders in a combination of different of types of patients with shock (Monnet et al., 2013). However, they did find a significant increase of ScvO_2_ from 64% ±4% to 71% ±2% in a subgroup of fluid responders, whose mean VO_2_ only increased by 5 ml/min. In comparison, in another subgroup of fluid responders whose VO_2_ increased by more than 15%, ScvO_2_ did not change significantly. Similarly, in a study performed in 40 septic shock patients, Xu et al showed that ΔScvO2 correlated well with ΔCI induced by fluid challenge when most of the patients (38/40) had a no increase of VO_2_ immediately after fluid challenge (Xu et al., 2017). Thus, it is conceivable that if VO_2_ increased significantly after fluid challenge, the correlation between ΔScvO_2_ and ΔCO would be compromised (Squara, 2014).

**TABLE 4:** Hemodynamic parameters. Values are expressed as mean ±SD

	Baseline	PLR	Preinfusion	After VE
VTI, cm				
Responders	15.1 ± 3.6	19.3 ± 4.3^a^	15.7 ± 4.4	20.7 ± 5.8^a^
Nonresponders	15.9 ± 4.3	16.5 ± 4.1^b^	16.2 ± 4.1	16.8 ± 4^b^
HR, b.p.m.				
Responders	75 ± 8	74 ± 12	76 ± 9	73 ± 11
Nonresponders	81 ± 10	79 ± 11	81± 9	79 ± 8
MAP, mmHg				
Responders	68 ± 9	80 ± 12^a^	69 ± 8	83.8 ± 10^a^
Nonresponders	72 ± 11	76 ± 11^a^	73 ± 11	79 ± 11^a^
CVP, mmHg				
Responders	7 ± 3	10 ± 2^a^	7 ± 3	11 ± 3^a^
Nonresponders	9 ± 4	11 ± 5^a^	9 ± 5	10 ± 4
ScvO_2_, (%)				
Responders	63 ± 10	68 ± 10^a^	64 ± 10	71.3 ± 10^a^
Nonresponders	67 ± 6	68 ± 7	67 ± 6	68 ± 6

VTI, Velocity Time Integrale; HR, Heart Rate; MAP, Mean Arterial Pressure; CVP, Central Venous Pressure; ScvO_2_, Central venous Oxygen Saturation; PLR, Passive Leg Raising; VE, Volume Infusion.

^a^ *p *< 0.05 versus baseline/preinfusion.

^b^ *p* < 0.05 versus nonresponders.

Response of SvO_2_ to PLR can be quickly observed when a SvO_2_ monitor is connected to a continuous spectrophotometer‐like CeVOX probe (PV2022‐37; Pulsion Medical System, Getinge, Munich, Germany). The present monitor has been well studied and the continuous estimation of ScvO_2_ was found both accurate and precise following calibration (Herner et al., 2018). However, the continuous technique used to measure SvO_2_ during PLR must be able to detect short‐term and transient changes in SvO_2_ since the PLR effects may vanish after 1 minute (Monnet & Teboul, 2015). In this regard, the present CeVOX in vivo fiber‐optic probe measure SvO_2_ with a resolution of around one percent. Moreover, SvO_2_ value is continuously, in real time, displayed by the oximetry monitor with an information processed, updated and displayed every few seconds as a percent value.

Another “sine qua none” condition of the utmost importance that may fundamentally affects CeVOX probe ability to measure changes in SvO_2_ following a PLR maneuver is the time reaction of SvO_2_ during acute change in blood flow induced by the procedure. In this regard, in a prospective cohort study, rapid ventricular pacing episodes (which, as PLR, affect cardiac preload) induced an abrupt drop in SvO_2_ with a rapid recovery following the arrest of the procedure. Moreover, the time reaction of SvO_2_ during change in blood flow induced by the procedure was of at least as long as 1 minute (Musialowicz et al., 2019).

The current study acknowledges some limitations. First, its relatively small and homogeneous sample size may require to be careful in interpreting these results to all ICU patients. Second, the majority of the patients were deeply sedated and mechanically ventilated with stable oxygen consumption. Third, two patients presented no increase in CVP value despite VE; this is a fact that may presume the occurrence of a capillary leak phenomenon, which is a pathophysiological condition that may affect the negative predicative value (i.e., a potential responder may be classified as a nonresponder) (Bendjelid, 2005). Finally, the perioperative state of cardiac surgical patients with perioperative hemodilution and consumption of coagulation factors, leads to a singular coagulopathy status that may improve continuous ScvO_2_ monitoring (no clots on the fiber‐optic probe).

## 5 CONCLUSION

ScvO_2_ variation induced by PLR is a reliable, minimally invasive parameter for predicting FR at the bedside of deeply sedated, mechanically ventilated, critically ill patients undergoing cardiac surgery.

## ETHICS APPROVAL AND CONSENT TO PARTICIPATE

Ethical approval for this study (serial number NAC 11–018) was obtained from the ethics committee of the Geneva University Hospitals. Oral and written informed consent were obtained from the patient or legal guardian.

## COMPETING INTERESTS

The authors declare that they have no competing interests.

## DATA AVAILABILITY STATEMENT

The datasets used and analyzed during the current study are available from the corresponding author on reasonable request.

## AUTHOR CONTRIBUTIONS

RG: conception and design of the work; acquisition, analysis, and interpretation of data; writing original draft. BV: acquisition, analysis, and interpretation of data; substantial revision of the manuscript. BA: interpretation of data; substantial revision of the manuscript. INS: analysis, and interpretation of data; substantial revision of the manuscript. KB: conception and design of the work; acquisition, analysis, and interpretation of data; writing original draft. All authors have approved the submitted version (and any substantially modified version that involves the author's contribution to the study), and have agreed both to be personally accountable for the author's own contributions and to ensure that questions related to the accuracy or integrity of any part of the work, even ones in which the author was not personally involved, are appropriately investigated, resolved, and the resolution documented in the literature. All authors read and approved the final manuscript.

## Supporting information



**Figure S1**.
**Figure S2**.
