# Supplementary material for: Do ScvO2 variations induced by passive leg raising predict fluid responsiveness? A prospective study
Source: Physiol Rep. 2021 Sep 7;9(17):e15012. doi: 10.14814/phy2.15012 (PMC8422598; doi:10.14814/phy2.15012)
Supplement: Supplementary file 1 — Figure S1. Figure S2. [file PHY2-9-e15012-s001.pptx]

## Slide 1
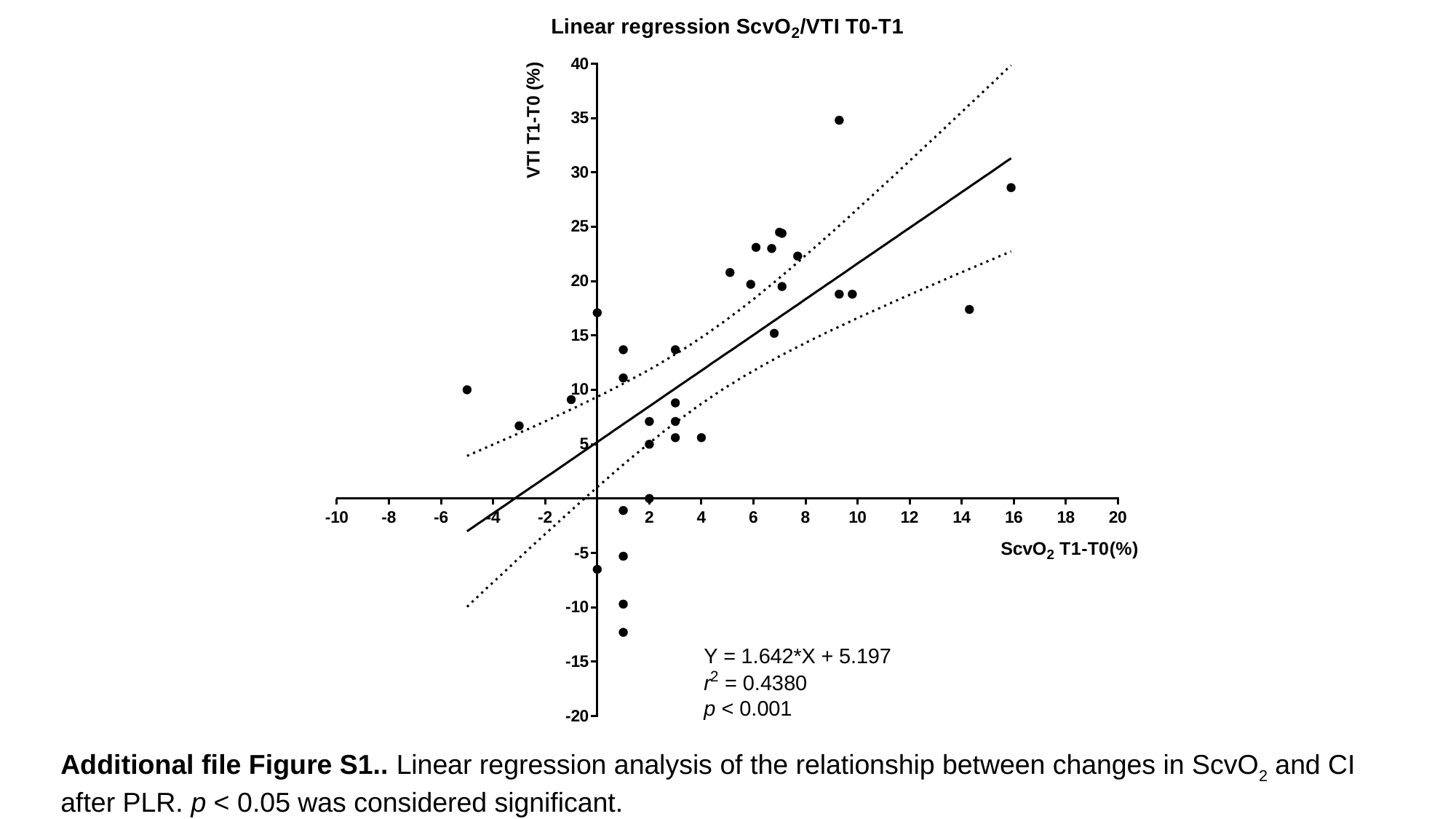

Additional file Figure S1.. Linear regression analysis of the relationship between changes in ScvO2 and CI after PLR. p < 0.05 was considered significant.

## Slide 2
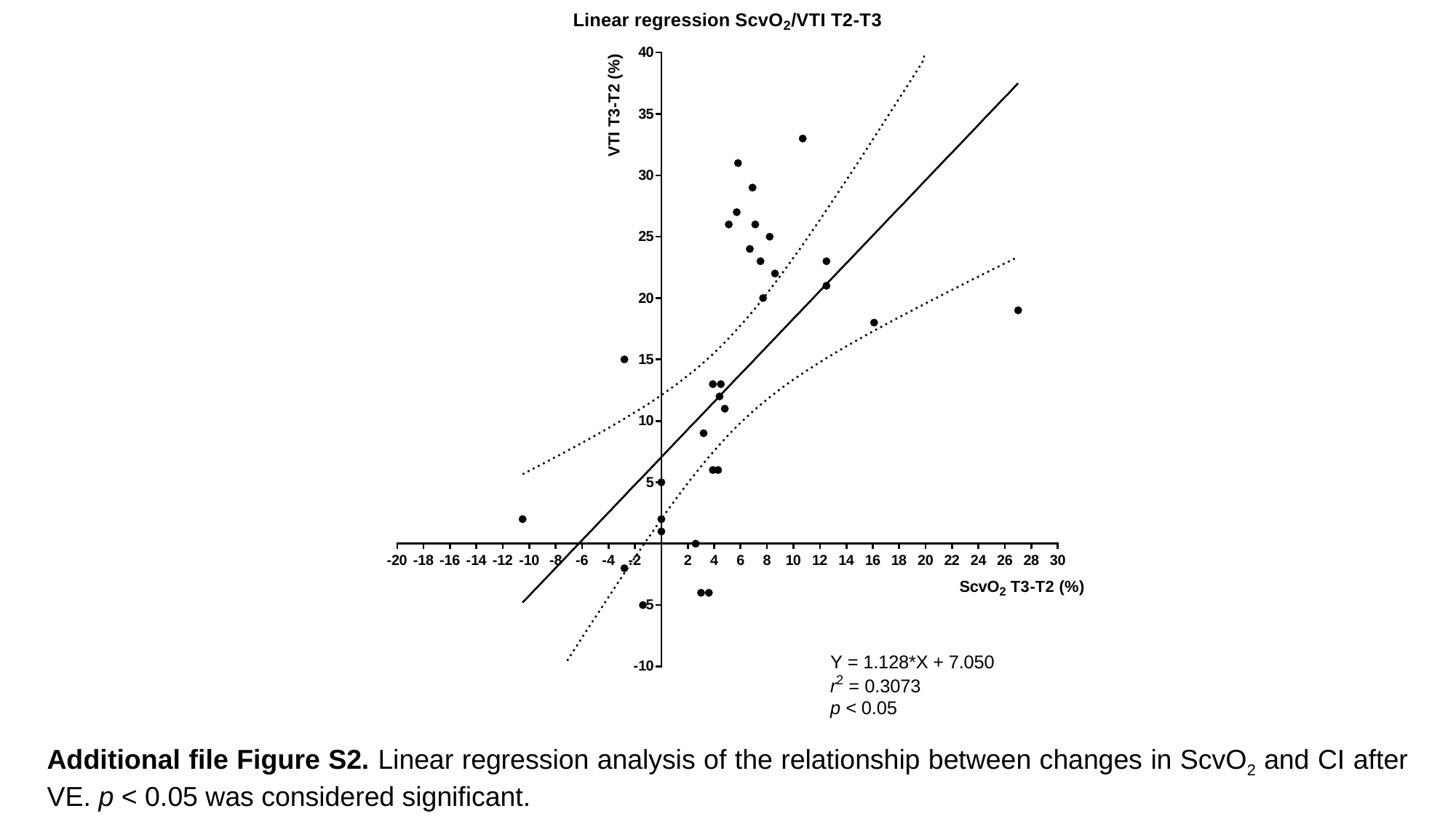

Additional file Figure S2. Linear regression analysis of the relationship between changes in ScvO2 and CI after VE. p < 0.05 was considered significant.
